# Comparative transcriptome analysis reveals the regulatory networks of cytokinin in promoting the floral feminization in the oil plant *Sapium sebiferum*

**DOI:** 10.1186/s12870-018-1314-5

**Published:** 2018-05-30

**Authors:** Jun Ni, Faheem Afzal Shah, Wenbo Liu, Qiaojian Wang, Dongdong Wang, Weiwei Zhao, Weili Lu, Shengwei Huang, Songling Fu, Lifang Wu

**Affiliations:** 1 Key laboratory of high magnetic field and Ion beam physical biology,Hefei Institutes of Physical Science, Chinese Academy of Sciences, Hefei, Anhui 230031 China; 2 School of Forestry and Landscape Architecture, Anhui Agricultural University, Hefei, Anhui China

**Keywords:** 6-BA, TDZ, Floral feminization, Bioenergy plant, *Sapium sebiferum*

## Abstract

**Background:**

*Sapium sebiferum*, whose seeds contain high level of fatty acids, has been considered as one of the most important oil plants. However, the high male to female flower ratio limited the seed yield improvement and its industrial potentials. Thus, the study of the sex determination in *S. sebiferum* is of significant importance in increasing the seed yield.

**Results:**

In this study, we demonstrated that in *S. sebiferum*, cytokinin (CK) had strong feminization effects on the floral development. Exogenous application with 6-benzylaminopurine (6-BA) or thidiazuron (TDZ) significantly induced the development of female flowers and increased the fruit number. Interestingly, the feminization effects of cytokinin were also detected on the androecious genotype of *S. sebiferum* which only produce male flowers. To further investigate the mechanism underlying the role of cytokinin in the flower development and sex differentiation, we performed the comparative transcriptome analysis of the floral buds of the androecious plants subjected to 6-BA. The results showed that there were separately 129, 352 and 642 genes differentially expressed at 6 h, 12 h and 24 h after 6-BA treatment. Functional analysis of the differentially expressed genes (DEGs) showed that many genes are related to the hormonal biosynthesis and signaling, nutrients translocation and cell cycle. Moreover, there were twenty one flowering-related genes identified to be differentially regulated by 6-BA treatment. Specifically, the gynoecium development-related genes *SPATULA* (*SPT*), *KANADI 2* (*KAN2*), *JAGGED* (*JAG*) and *Cytochrome P450 78A9* (*CYP79A9*) were significantly up-regulated, whereas the expression of *PISTILLATA* (*PI*), *TATA Box Associated Factor II 59* (*TAFII59*) and *MYB Domain Protein 108* (*MYB108*) that were important for male organ development was down-regulated in response to 6-BA treatment, demonstrating that cytokinin could directly target the floral organ identity genes to regulate the flower sex.

**Conclusions:**

Our work demonstrated that cytokinin is a potential regulator in female flower development in *S. sebiferum*. The transcriptome analysis of the floral sex transition from androecious to monoecious in response to cytokinin treatment on the androecious *S. sebiferum* provided valuable information related to the mechanism of sex determination in the perennial woody plants.

**Electronic supplementary material:**

The online version of this article (10.1186/s12870-018-1314-5) contains supplementary material, which is available to authorized users.

## Background

*Sapium sebiferum*, a member of the Euphorbiaceae family, is one of the most important perennial oil plants. It has attracted great attention due to its high oil production, pharmaceutical values, high ornamental values as landscape plant and its viability to grow in the marginal land (Peng et al. 2008; Yang et al., 2007). *S. sebiferum* has been cultivated as a valuable oil plant for more than 14 centuries, due to the high oil content of the seed coat and seed kernel, which can be used for the manufacture of industrial products, such as lubricant, soap and candles [1]. Aside from being a source for industrial purposes, the oil extracted from the *S. sebiferum* is also considered as a kind of renewable oil resources, which can be further exploited for the production of diesel fuel and fatty acid alkyl esters [2, 3]. Nevertheless, low female to male flower ratio significantly limited the seed yield improvement and the industrial potentials of *S. sebiferum*. Most of the *S. sebiferum* plants are monoecious with separate male and female flowers on the same inflorescence, while some are androecious plants that only produce male flowers. There are over one hundred male flowers arrange in the narrow raceme-like inflorescence, while only with few female flowers growing at the base of the inflorescence. Thus, the manipulation of the flower sex differentiation by using the biochemical and genetic strategies was of significant importance in improving the seed yield in *S. sebiferum*.

Phytohormones are essential factors involved in the regulation of vegetative and reproductive growth in the plant kingdom. Phytohormones, such as cytokinin (CK), auxin, gibberellin (GA) and ethylene (ETH), were demonstrated to be involved in the regulation of flowering and sex determination in many species [4]. Generally, cytokinin, auxin and ethylene were considered as positive regulators in gynoecium development, whereas GA (gibberellin) promoted the development of androecious organs [5–8]. In *Jatropha curcas* and *Plukenetia volubilis*, exogenous application with cytokinin can increase the female flower number [9, 10]. Nevertheless, the mechanisms how cytokinin regulated the sex determination remains largely unknown. In this study, we demonstrated that CK was a key regulator in sex determination in the oil plant *S. sebiferum*. CK treatment on the floral bud significantly induced the floral feminization and promoted the fruiting. As the seed yield improvement of *S. sebiferum* was greatly limited by the low female to male flower ratio, thus our findings will be of significant importance in the cultivation of *S. sebiferum*. Further, the feminization effect was also detected on the androecious genotype of *S. sebiferum*. To further investigate how CK regulated the transition of the floral bud from androecious to monecious, RNA-seq analysis was carried out on the floral buds of the androecious *S. sebiferum* at 6 h, 12 h and 24 h after 6-BA treatment. The present study not only provided an effective method in improving the seed yield during *S. sebiferum* cultivation, the transcriptome data also provided insights in understanding the mechanism of sex determination regulated by cytokinin in the woody plants.

## Methods

### Plant materials and growth conditions

In this experiment, three-year-old *S. sebiferum* trees, growing in the experimental field in Hefei Institutes of Physical Science (N 31° 49′, E117° 13′), Chinese Academy of Sciences, were used for the hormonal treatment. Trees were planted with 2.5 m × 2.5 m spacing, and fertilized every year before bud sprouting.

### Phytohormone preparation and application

To make stock solutions (20 mM) of phytohormones, 6-BA, TDZ, trans-zeatin (tZ) or kinetin was dissolved in 0.2 M NaOH solution. The stock solutions were diluted with water to make different concentrations of working solutions containing 0.05% Tween-20 [11]. All working solutions for each treatment had the same solvent. The floral buds (one week after its formation) were treated with working solutions using a 100 ml plastic sprayer.

### Sample collection and RNA isolation for transcriptome sequencing and quantitative real time PCR

Floral buds of the androecious plants (Additional file 1: Figure S1) were treated with 6-BA (200 μM) and mock solution (containing the same solvent with 6-BA solution). The floral buds after removing the leaves (approximately 3 mm in length, Additional file 1: Figure S1) were collected at 6 h, 12 h and 24 h separately, and then immediately frozen in liquid nitrogen. Each treatment at each time point was prepared with six independent biological replicates (three for RNA seq, and three for quantitative real time PCR). The total RNA was extracted by using a Plant Total RNA Isolation Kit (Omega, China). RNA purity was checked by gel electrophoresis and NanoDrop 2000c spectrophotometer (Thermo Fisher, MA, USA). RNA concentration and integrity were separately determined using a Qubit 2.0 Flurometer (Life Technologies, Carlsbad, CA, USA) and Agilent Bioanalyzer 2100 system (Agilent Technologies, Santa Clara, CA, USA).

### Transcriptome sequencing and de novo transcriptome assembly

Sequencing libraries (each sample with three biological replicates) were prepared following the instructions of the NEBNext Ultra RNA Library Prep Kit for Illumina (NEB, USA). The literary quality was determined using the Agilent Bioanalyzer 2100 system. The clustering of the index-coded samples was performed according to the instructions of cBot Cluster Generation System. Then the libraries were sequenced on the Illumina HiSeq 2000 platform at the Beijing Genomics Institute (BGI, Shenzhen, China) and the raw reads were generated in 90-bp paired-end format. The low-quality reads (< 20 Phred scores) were removed by Fastq_clean [12] and further assessed by FastQC [13]. The clean reads were assembled by using Trinity (version 2.0.6) [14]. The paired-end reads were then mapped to the de novo assemblies by using Bowtie (version 1.1.1) [15]. The abundance estimation was performed using Corset (version 1.03) [16].

### Transcript annotation and gene expression analysis

All the assembled transcripts were searched against the NCBI non-redundant protein database (Nr), Swiss-Prot protein database and NCBI non-redundant nucleotide sequence (Nt) database. The GO functional classification was then carried out using Blast2GO program based on the Nr annotation [17]. The GO analysis was carried out by using the WEGO software [18]. The transcripts were aligned to the Clusters of Orthologous Groups of proteins (COG) database and Kyoto Encyclopedia of Genes and Genomes (KEGG) database [19, 20]. To quantify the differentially expressed unigenes, the read counts were calculated by using the fragments per kilobase of transcript per million fragments mapped method (FPKM) [21].

### Quantitative real time PCR (qPCR) analysis

cDNA was synthesized according to the instructions of EasyScript One-Step gDNA removal and cDNA synthesis SuperMix (Transgen Biotechnology, Beijing, China). qPCR was performed using TaKaRa SYBR Premix Ex Taq™ II (TaKaRa Biotechnology, Dalian, China) on the Roche Light Cycler 96 System (Roche, Swiss). The PCR procedures were as follows: 95 °C for 5 min; 45 cycles of 95 °C for 10 s, 60 °C for 10 s, and 72 °C for 15 s; the melting curve analysis was from 65 °C to 95 °C. The information of the primers was listed as Additional file 2: Table S1.

### Statistical analyses

Data were analyzed using the Statistical Product and Service Solution (SPSS) version 13.0 software. The significance between the hormone treated and control group was determined using Student’s test.

## Results

### 6-BA and TDZ had strong feminization effects on the floral development and promoted fruiting in *S. sebiferum*

**Fig. 1:**
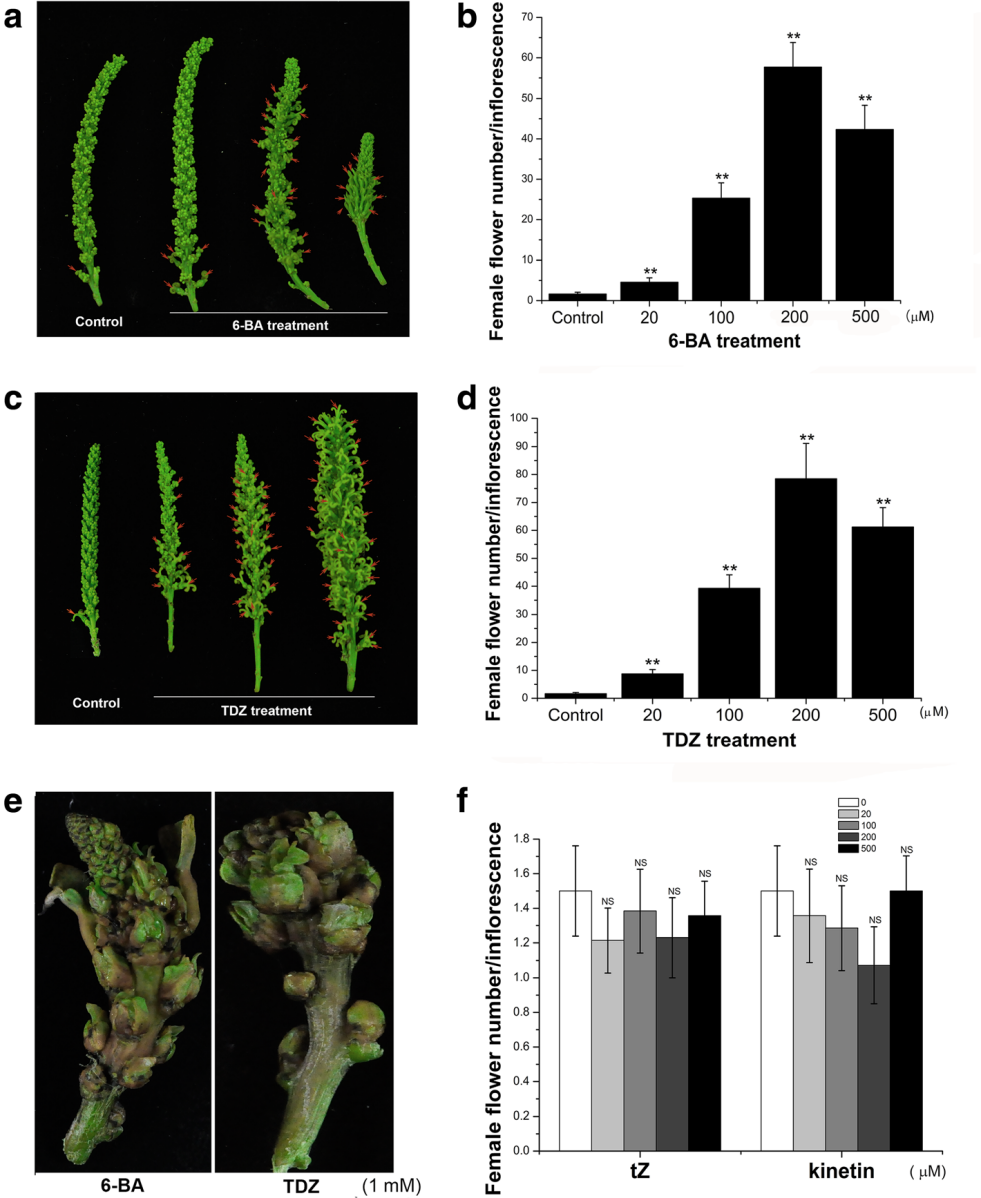
Effects of 6-BA and TDZ on the induction of female flowers in *S. sebiferum*. **a** Inflorescences of control and 6-BA-treated groups. **b** Number of female flowers per inflorescence three weeks after 6-BA treatment (*n* = 30). **c** Inflorescences of control and TDZ-treated groups. **d** Number of female flowers per inflorescence by TDZ treatment (n = 30). **e** Inflorescences of 1 mM 6-BA or TDZ treated groups. **f** Number of female flowers per inflorescence by tZ and kinetin treatment (n = 30). Values are mean ± SE. Significance between treatment and control was determined by Student’s t test. Significance level: ^**^P < 0.01. NS, no significance. Red arrows indicated the induced female flowers

**Fig. 2:**
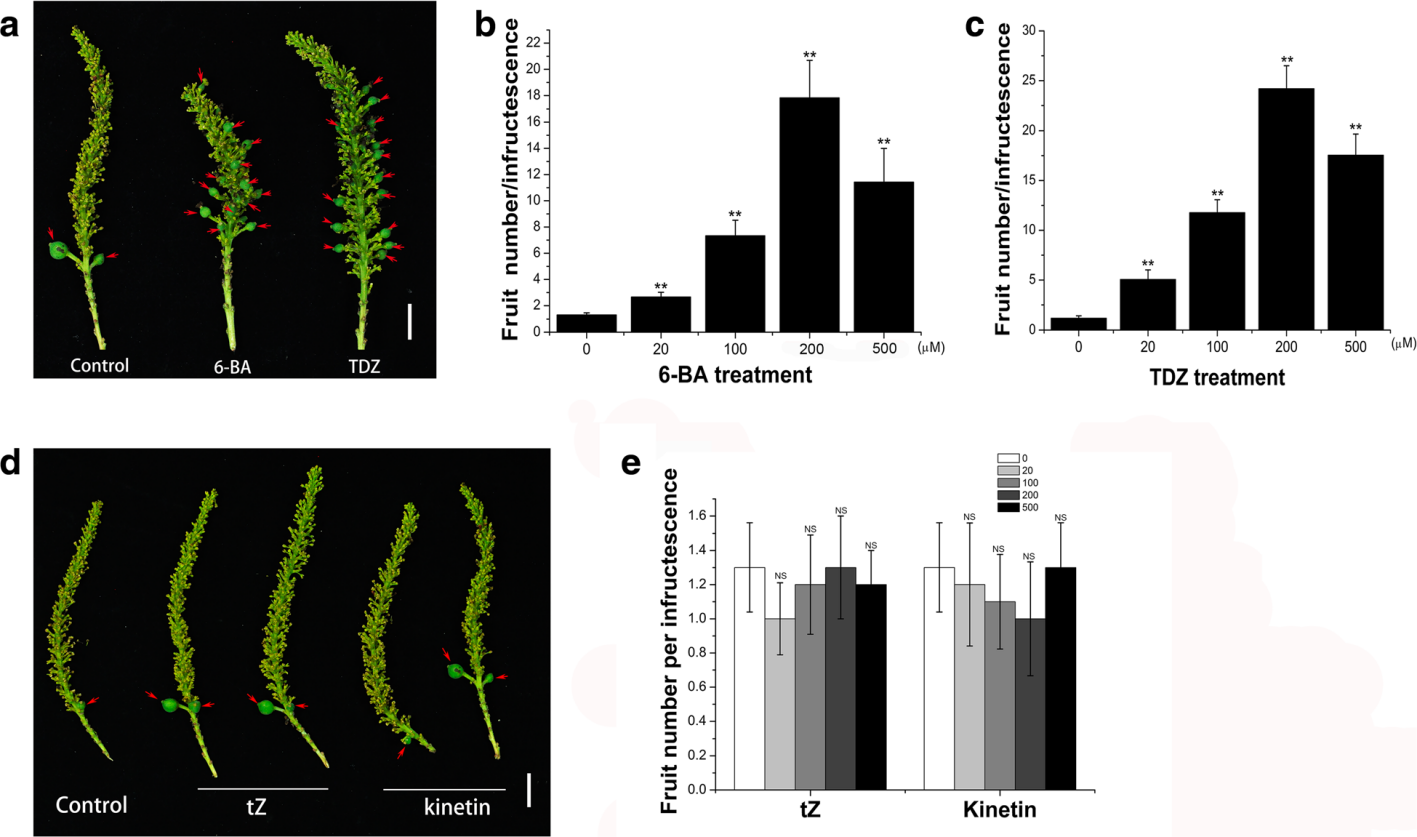
6-BA and TDZ promoted fruiting in *S. sebiferum*. **a** Fructescence of 6-BA and TDZ treated groups. **b** and **c** Fruit number was counted approximately five weeks after 6-BA or TDZ treatments (n = 30). **d** Fructescence of tZ and kinetin treated groups. **e** Fruit number per infructescence by tZ and kinetin treatment (n = 30). Values are mean ± SE. Significance between treatment and control was determined by Student’s t test. Significance level: ^**^P < 0.01. NS, no significance

Based on the floral structure, *S. sebiferum* can be mainly divided into two main genotypes, *S. sebiferum* var. conferticarpa and *S. sebiferum* var. laxiarpa [22]. Most of *S. sebiferum* var. conferticarpa plants are monoecious, with few female flowers growing at the floral base, while some are androecious plants that only produce male flowers (Additional file 1: Figure S1). The results showed that exogenous 6-BA or TDZ treatment significantly induced the female flowers, which appeared at the position where the male flowers located (Fig. 1). The feminization effect was increased with increasing concentrations of 6-BA (Fig. 1a, b), or TDZ (Fig. 1c, d), reaching an average of 57.7 and 78.5 female flowers per inflorescence, separately by 6-BA and TDZ treatment. TDZ treatment had better effects in promoting the floral development, resulting in larger floral size and higher female flower number than that by 6-BA treatment (Fig. 1). However, over-dosed 6-BA or TDZ (e.g. 1 mM) was actually toxic to the floral bud (Fig. 1e). Approximately 20–30% of the 6-BA- or TDZ-induced female flowers could further develop into fruits (Fig. 2). It was worthy to mention here that although trans-zeatin (tZ) or kinetin were two other types of cytokinins. Exogenous application with these tZ or kinetin had no effects on the flower development or fruiting in *S. sebiferum* (Figs. 1f and 2d, e).

### 6-BA or TDZ treatment feminized the androecious genotype of *S. sebiferum*

**Fig. 3:**
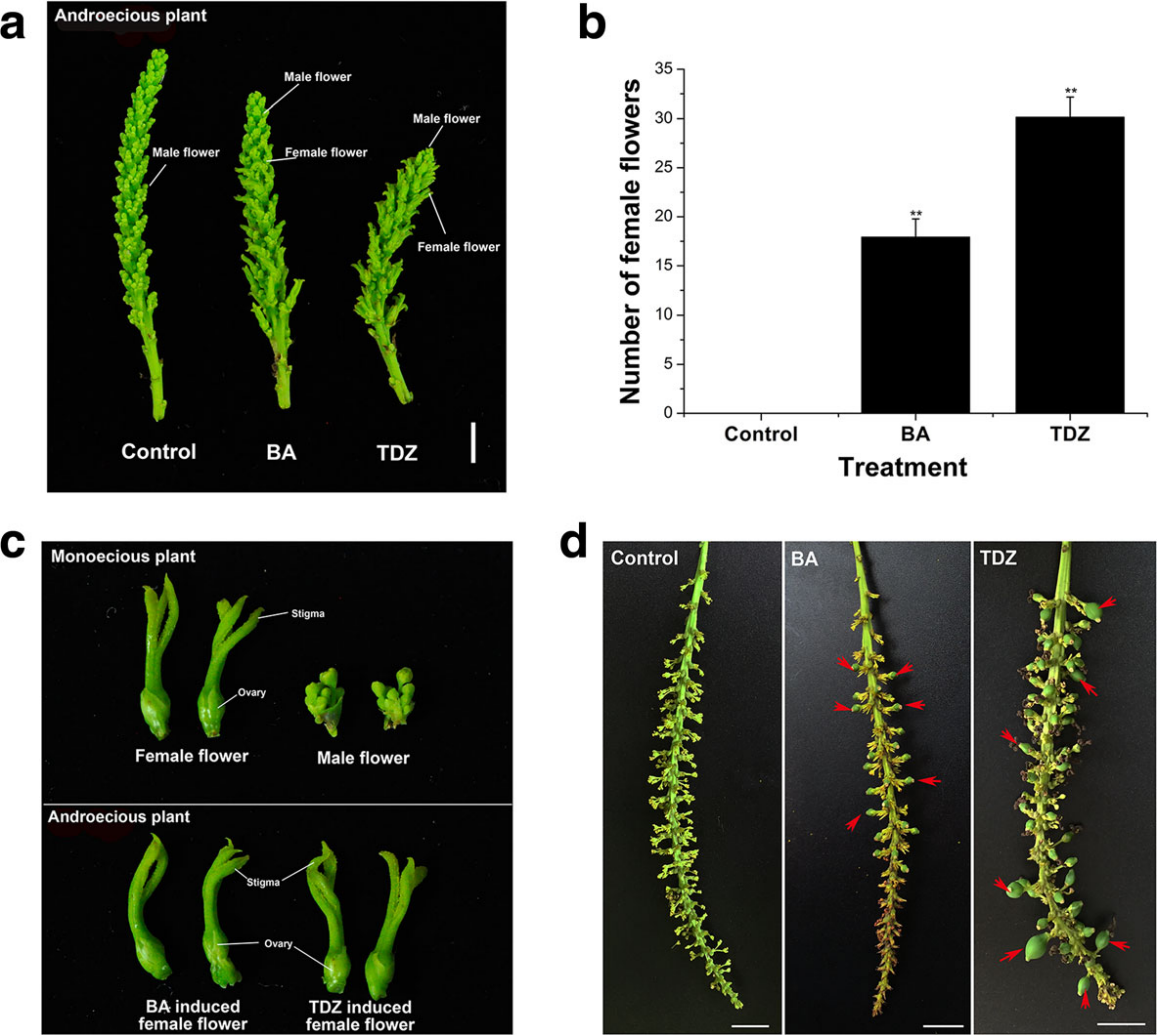
6-BA or TDZ induced female flower development on the androecious *S. sebiferum*. **a** Inflorescences of control, 6-BA- and TDZ-treated *S. sebiferum*. **b** Number of female flowers was counted after 6-BA or TDZ treatment (100 μM) (n = 30). **c** 6-BA- or TDZ-induced female flowers on the androecious plants showed similar phenotype with that of the monoecious *S. sebiferum*. **d** Infructescences of the androecious plants after control, 6-BA- or TDZ treatment. Values are mean ± SE. Significance between treatment and control was determined by Student’s t test. Significance level: ^**^P < 0.01. Bars = 1 cm

In *S. sebiferum*, there are a small population of androecious plants that only produces male flowers and bares no fruits (Additional file 3: Figure S2). The effects of cytokinin on the floral development on the androecious genotype of *S. sebiferum* was also investigated in this study. The results showed that 6-BA or TDZ treatment can induce the female flower development on the male inflorescence of the androecious *S. sebiferum* (Fig. 3a, b). The induced female flowers, which had similar structures with that of the normal flowers (Fig. 3c), were located at the position of the male flowers all over the whole inflorescence, and can further develop into fruits (Fig. 3d).

### Transcriptome sequencing of the floral buds of the androecious *S. sebiferum* in response to 6-BA treatment

The androecious genotype provided great advantages in investigating the mechanism of sex differentiation regulated by cytokinin in *S. sebiferum*. After 6-BA or TDZ treatment, abundant female flowers were induced in the “male inflorescence”, thus we presumed that cytokinin was not only the key factor in regulating the sex differentiation in *S. sebiferum*, but also possibly participated in the formation of the *androecious* plants. The transcriptome analysis of the floral buds at the early developing stages of the androecious plants would be helpful for identification of key genes involved in the female flower formation and development in response to cytokinin in *S. sebiferum*.

**Fig. 4:**
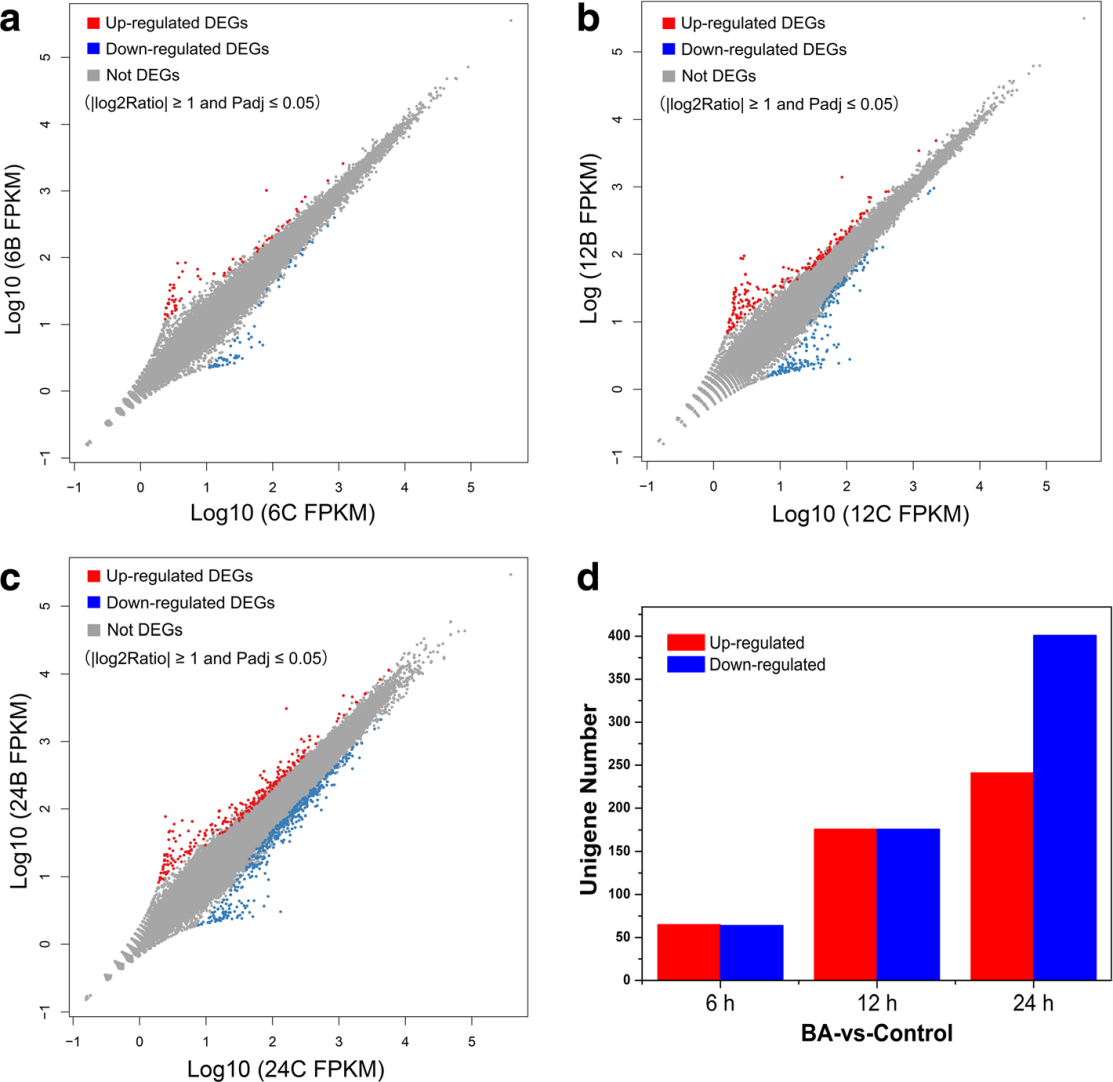
Differentially expressed genes at 6 h, 12 h and 24 h after 6-BA treatment on the floral buds of the androecious *S. sebiferum*. **a-c**. The differential gene expression was plotted using the FPKM method. The DEGs with |log2 ratio| ≥ 1 and Padj≤0.05 were shown in red and green dots. **d** Number of DEGs. Red and green columns represented the number of up-, or down-regulated DEGs, respectively

**Fig. 5:**
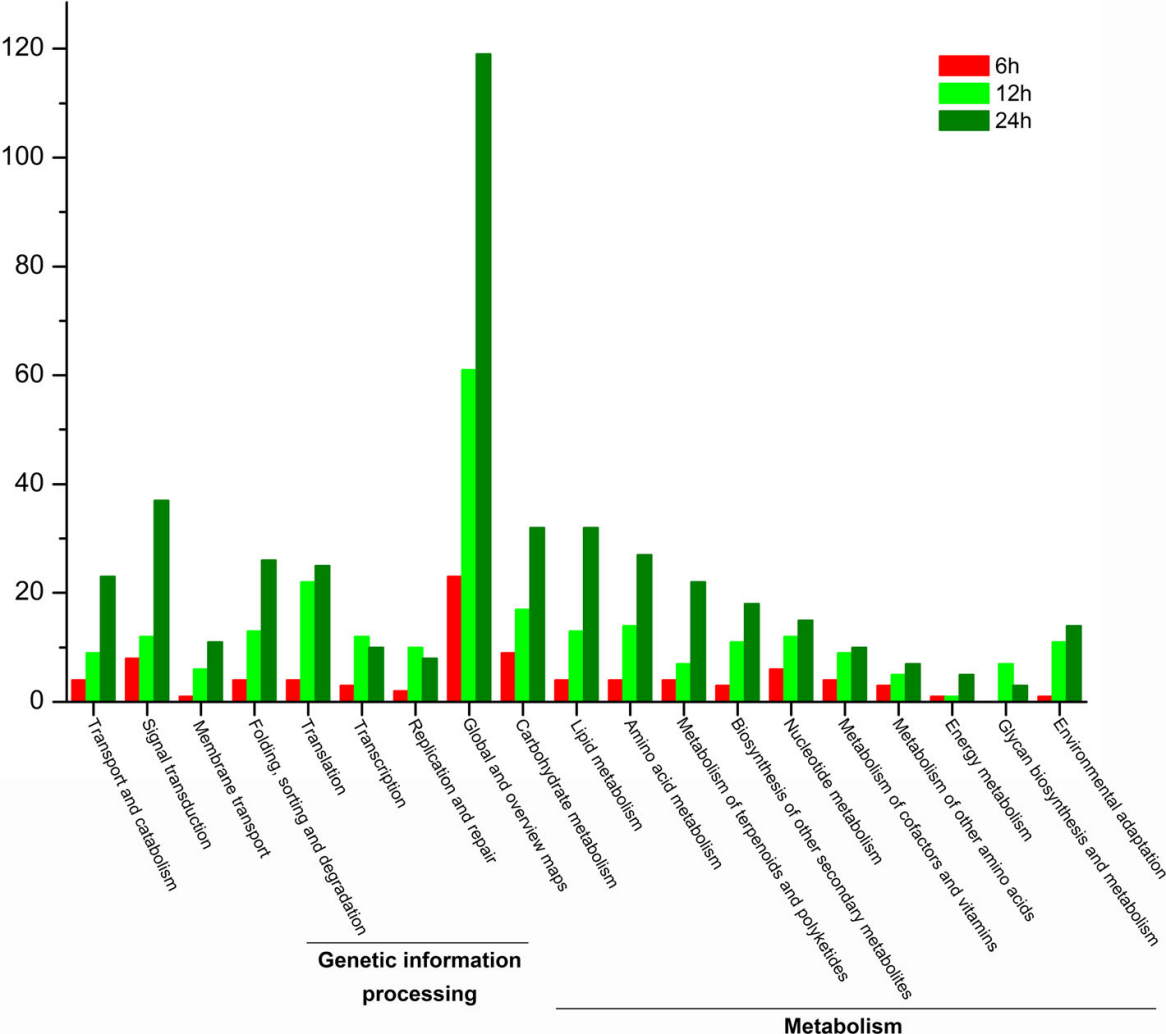
KEGG analysis of the differentially expressed genes at 6 h, 12 h and 24 h after 6-BA treatment

In *S. sebiferum*, the floral buds were located at the terminal of each branchlet. One week after the floral bud formation (approximately 3 mm in length, Additional file 1: Figure S1), 6-BA (200 μM) and mock solutions were directly applied to the floral buds. In total of six groups of samples (6-BA- and mock-treated floral buds at 6 h, 12 h and 24 h, each group contains six biological replications) were collected for the following transcriptome sequencing and quantitative real-time PCR, aiming at identification of the early responsive genes that were involved in the sex determination regulated by cytokinin. Eighteen cDNA libraries were constructed and sequenced on the Illumina high-throughput sequencing platform. The detailed information of the transcriptome assembly can be found in Table 1 and Additional file 4: Tables S2-S4. The differences of gene expression levels were plotted with colored dots and there were separately 129, 352 and 642 genes differentially expressed at 6 h, 12 h and 24 h after 6-BA treatment (Fig. 4, Additional file 5: Tables S5-S7). To validate the accuracy of the transcriptome sequencing results, the expression level of fourteen differentially expressed genes (DEGs) was determined by quantitative real-time PCR (qPCR). The expression profiles of these genes by qPCR were consistent with the values derived from the RNA-seq (Table 2). Kyoto Encyclopedia of Genes and Genomes (KEGG) and Gene Ontology (GO) analysis were further carried out to generally describe the biological functions of these differentially expressed genes (DEGs) (Fig. 5 and Additional file 3: Figure S2). The results showed that the DEGs were mainly classified into 19 categories by KEGG analysis and the predominant categories were mostly related to “Metabolism” (58%) and “Genetic information processing” (21%) (Fig. 5). Further, the predominant categories by GO analysis were “metabolic process”, “cellular process”, “cell”, and “catalytic activity” (Additional file 6: Figure S3), which was closed correlated to the basic biological functions of cytokinin in promoting cell growth, replication and metabolism.

**Table 1:** Summary of the transcriptome assembly

Sample	Total Number	Total Length (nt)	Mean Length (nt)	N50	N70	GC(%)
6C-1	78,134	72,513,692	928	1585	988	40.19
6C-2	83,602	82,610,570	988	1709	1073	39.76
6C-3	75,035	71,403,325	951	1645	1026	40.05
6BA-1	71,419	68,445,465	958	1648	1039	40.33
6BA-2	85,666	84,464,445	985	1709	1076	39.71
6BA-3	81,033	77,824,709	960	1662	1041	39.85
12C-1	80,069	77,792,164	971	1684	1062	39.91
12C-2	79,399	77,063,614	970	1670	1061	40.01
12C-3	77,903	74,154,878	951	1655	1036	40.03
12BA-1	77,472	74,784,107	965	1671	1050	40.09
12BA-2	78,727	75,826,676	963	1672	1051	40
12BA-3	77,233	74,834,430	968	1686	1058	40.01
24C-1	84,217	79,418,426	943	1608	1004	39.99
24C-2	69,973	64,954,222	928	1605	1001	40.26
24C-3	79,644	75,334,917	945	1637	1022	39.93
24BA-1	76,570	72,387,799	945	1645	1021	40.04
24BA-2	75,078	70,913,371	944	1635	1021	40.14
24BA-3	74,125	69,581,278	938	1629	1010	40.21

**Table 2:** Log2-fold change (6-BA vs Control at 6, 12 and 24 h) of the DEGs between RNA-seq and qPCR

Description		6 h		12 h		24 h	
Gene ID	Gene Name	RNA-seq	qPCR	RNA-seq	qPCR	RNA-seq	qPCR
CL3798.Contig10_All	Shaker Pollen Inward K+ Channel	0.2	−0.4	2.1	1.7	0.1	0.6
CL15451.Contig2_All	Cytochrome P450 78A9	0.6	0.3	1.1	1.6	1.1	0.9
CL14312.Contig3_All	RPB15.9.9	1.4	1.9	3.5	2.6	0.1	−0.4
CL10984.Contig2_All	YUCCA4	1.1	1.6	0.8	1.5	1.2	2.5
CL8402.Contig2_All	Fantastic Four 3-like	1.9	2.5	1.2	0.8	2.3	1.6
CL1856.Contig26_All	ATP-dependent DNA helicase	3.8	3.2	0.5	−0.3	0	0.5
CL1323.Contig24_All	Polygalacturonase	2.9	2.5	−1.3	− 1.6	0.7	0.2
CL14183.Contig4_All	FANCL isoform X2	0.3	0.1	−3.9	−2.6	0.2	−0.5
CL4361.Contig13_All	Casein kinase I	−0.2	0	−1.6	−2	0.6	0.2
CL4670.Contig3_All	Ethylene Response DNA Binding Factor 4	−1.1	−2.2	−0.5	−0.1	− 2	− 1.3
CL8816.Contig1_All	uncharacterized protein	−0.2	0.5	−0.15	0	3.9	2.8
CL2483.Contig2_All	β-1,3-galactosyltransferase 2	0.2	−0.4	0.4	−0.1	−3	−3.3
CL7852.Contig4_All	Response Regulator 5	1.2	2.4	0.9	1.5	1.4	2.6
CL14906.Contig2_All	Xyloglucan Endotransglycosylase	−1.2	−2.3	−1.1	−0.4	− 1.4	− 1

### Differentially expressed genes related to cell cycle and nutrient translocation

**Fig. 6:**
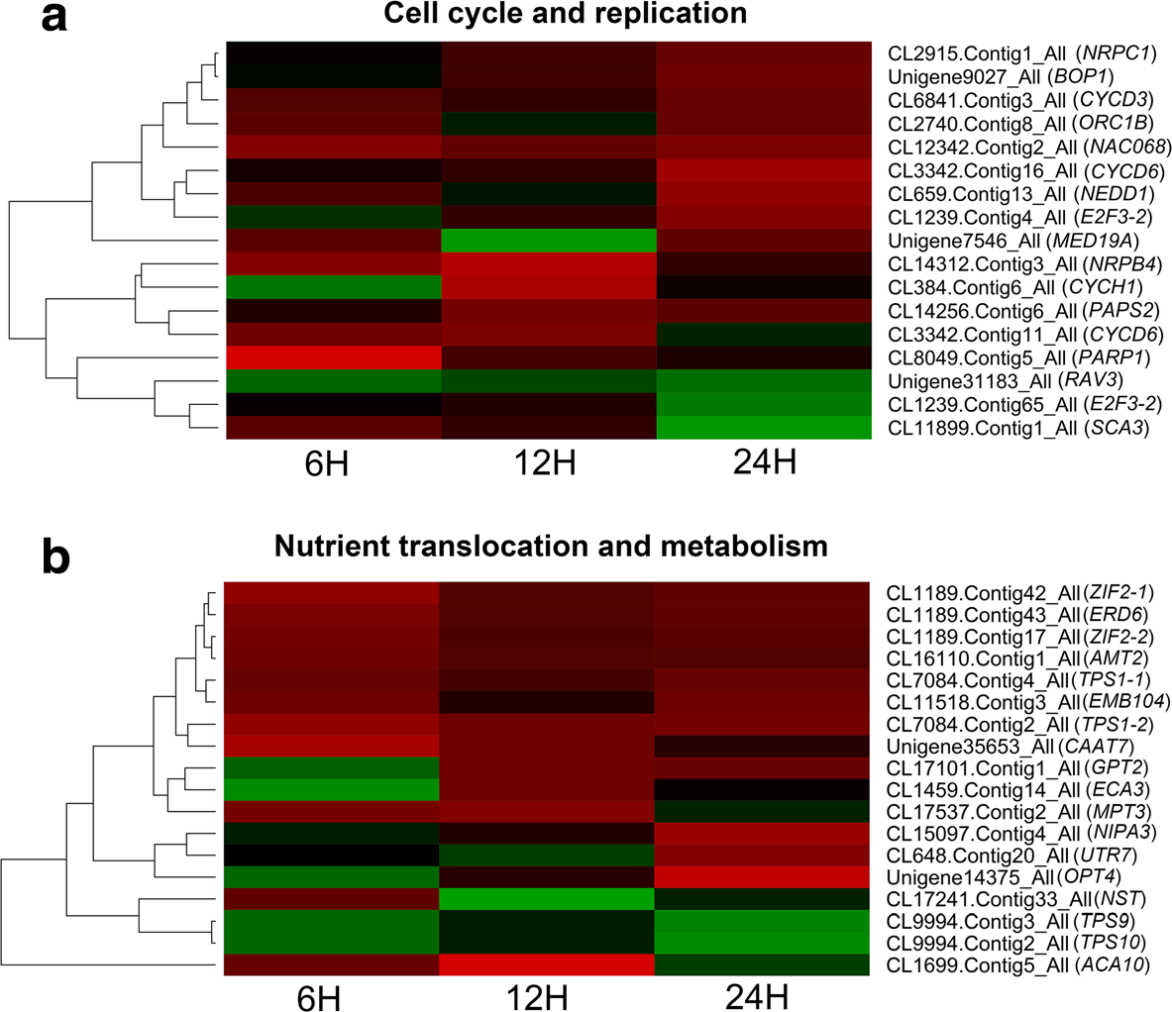
Hierarchical clustering of the differentially expressed genes related to cell cycle and nutrient translocation after 6-BA treatment. The expression profiles of the differentially expressed genes related to cell cycle and replication (**a**), and the translocation of sugar, oligopeptide, magnesium, ammonium, nucleotide, phosphate, calcium and zinc (**b**) were obtained at 6 h, 12 h and 24 h after 6-BA treatment

The KEGG analysis revealed that abundant DEGs were classified into “Metabolism” and “Genetic information processing” categories (Fig. 5). Many cell cycle related DEGs, including four cyclin genes, *CYCD3* (CL6841.Contig3_All), *CYCD6* (CL3342.Contig16_All and CL3342.Contig6_All), *CYCH1* (CL384.Contig6_All), and some other DEGs, such as *E2F Transcription factor 3* (*E2F3*, CL1239.Contig4_All), *ANAC068* (CL12342.Contig2_All), *Block of Cell Proliferation 1* (*BOP1*, Unigene9027_All), *NEDD1* (CL659.Contig13_All) and *Origin of Replication Complex 1B* (*ORC1B*, CL2740.Contig8_All) which encodes key transcription factors that involved in the regulation of cell division, were up-regulated after 6-BA treatment (Fig. 6a). Several DEGs that encoded DNA and RNA polymerases (Unigene7546_All, CL14256.Contig6_All, CL14312.Contig3_All and CL8049.Contig5_All) were also up-regulated in response to 6-BA treatment (Fig. 6a). In accompany with the enhanced cell division, the nutrients homeostasis in the floral bud was also affected by 6-BA treatment. The expression level of the nutrient translocation-related genes including *Early Response to Dehydration 6* (*ERD6*, CL1189.Contig43_All), *Glucose-6-Phosphate Translocator 2* (*GPT2*, CL17101.Contig1_All), *Nucleotide/Sugar Transporter* (*NST*, CL17241.Contig33_All) and *UDP-Galactose Transporter 7* (*UTR7*, CL648.Contig66_All) that involved in sugar transportation, *Oligopeptide Transporter 4* (*OPT4*, Unigene14375_All) in oligopeptide transportation, *Cationic Amino Acid Transporter 7* (*CAT7*, Unigene35653_All) in amino acid transportation, *ENOR3L5* (CL15097.Contig4_All) in magnesium transportation, *Ammonium Transporter 2* (*AMT2*, CL16110.Contig3_All) in ammonium transportation, *Embryo Defective 104* (*EMB104*, CL11518.Contig3_All) in nucleotide transportation, *Mitochondrial Phosphate Transporter 3* (*MPT3*, CL17537.Contig2_All) in phosphate transportation, two *Calcium Transporting ATPases ACA10* (CL1699.Contig5_All) and *ECA3* (CL1459.Contig14_All) in calcium transportation, and *Zinc-Induced Facilitator 2* (*ZIF2*, CL1189.Contiga42_All and CL1189.Contig17_All) in zinc transportation, were mostly up-regulated in response to 6-BA treatment (Fig. 6b). Recent studies demonstrated that trehalose-6-phosphate (T6P) was directly involved in the regulation of flowering, the biosynthesis of which was tightly regulated by the *Thehalose-6-Phosphate Synthase* (*TPS*) [23]. The transcriptome data also showed that four *TPS* genes, *TPS1* (CL7084.Contig2_All and CL7084.Contig4_All), *TPS9* (CL9994.Contig3_All) and *TPS10* (CL9994.Contig2_All), were differentially regulated by 6-BA treatment (Fig. 6b).

### Differentially expressed genes related to cytokinin metabolism and signaling

**Fig. 7:**
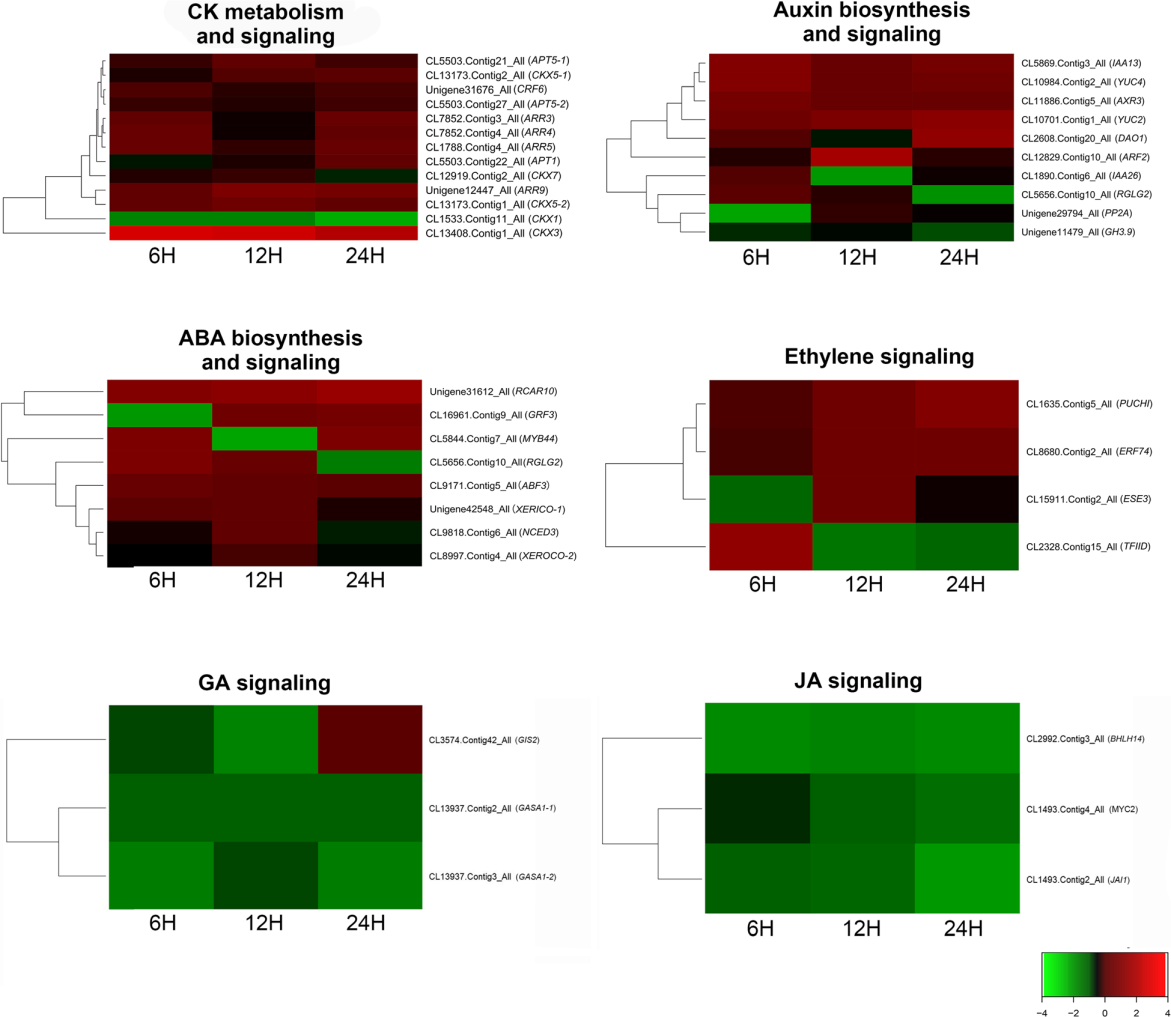
Hierarchical clustering of the differentially expressed genes related to the biosynthesis, metabolism and signaling of the phytohormones. The differentially expressed genes involved in the biosynthesis, metabolism or signaling of CK, auxin, ethylene, ABA, GA or JA were identified at 6 h, 12 h and 24 h after 6-BA treatment

After gene annotation, many cytokinin metabolism- and signaling-related genes were identified to be differentially expressed in response to 6-BA treatment (Fig. 7). *Adenine Phophoribosyltransferases* (*APT*s) and *Cytokinin Oxidase*s (*CKX*s) are two key regulators in maintaining the endogenous cytokinin homeostasis. APTs participated in the interconversion of cytokinin bases into nucleotides, while CKXs can directly catalyze the degradation of cytokinins [24, 25]. In this study, three *APT* genes *ATP1* (CL5503.Contig22_All), *APT5* (CL5503.Contig21_All and Cl5503.Contig27_All), and four *CKX* genes *CKX3* (CL13408.Contig1_All), *CKX5* (CL13173.Contig2_All and CL13173.Contig1_All), *CKX7* (CL12919.Contig2_All), were significantly up-regulated at 6 h, 12 h and 24 h after 6-BA treatment (Fig. 7), suggesting a strong feedback regulation of CK homeostasis after 6-BA treatment.

Previous studies suggested that cytokinin signal transduction occurs though a two-component phosphorelay system. *Arabidopsis response regulator*s (*ARR*s) can be classified into two groups (type A and type B), both of which are important components in cytokinin signal transduction [26]. Our results showed that the expression of four type A *ARR* genes, *ARR3* (CL7852.Contig3_All), *ARR4* (CL7852.Contig4_All), *ARR5* (CL1788.Contig4_All) and *ARR9* (Unigene12447), was up-regulated at 6 h, 12 h or 24 h after 6-BA treatment (Fig. 7). As the expression of type B *ARR*s was not significantly affected after 6-BA treatment, it suggested that in *S. sebiferum*, cytokinin signal transduction in the floral bud might be dependent on the regulation of type A *ARR*s.

### Differentially expressed genes related to hormonal biosynthesis, metabolism and signaling

Phytohormones, such as CK, auxin, ABA, ETH and GA are the key regulators in controlling floral initiation, development, and sex differentiation. We further investigated whether the biosynthesis and signaling of the other hormones were affected by 6-BA treatment. The results showed that three auxin metabolism- and six auxin signaling-related genes were differentially expressed at 6 h, 12 h or 24 h after 6-BA treatment (Fig. 7). *YUCCA2* (*YUC2)* and *YUCCA2* (*YUC4*) which encoded two YUC flavin monooxygenases, played essential roles in auxin biosynthesis [27]. In *S. sebiferum*, both *YUC2* (CL10701.Contig1_All) and *YUC4* (CL10984.Contig2_All) was up-regulated at 12 h and reached a higher expression level at 24 h after 6-BA treatment (Fig. 7). Meanwhile, the expression of *Dioxygenase for Auxin Oxidation 1* (*DAO1*, CL2608.Contig20_All), which encoded a key auxin oxidase and was also a major contributor to IAA degradation [28], was firstly decreased at 12 h, then increased at 24 h after 6-BA treatment (Fig. 7), suggesting there could be a transient increase of endogenous auxin level after 6-BA treatment. Further, three ABA biosynthesis- and five ABA signaling-related genes were also regulated by 6-BA treatment. The expression of ABA biosynthesis-related genes *XERICO* (Unigene42548_All and CL8997.Contig4_All) and *NCED3* (CL9818.Contig6_All) was significantly down-regulated by 6-BA treatment (Fig. 7). Four ABA signaling-related genes *Regulatory Components of ABA Receptor 10* (*RCAR10*, Unigene31612_All), *Ring Domain Ligase 2* (*RGLG2*, CL5656.Contig10_All), *Growth-regulating Factor 3* (*GRF3*, CL16961.Contig9_All) and A*bscisic Acid Responsive Elements*-*binding Factor 3* (*ABF3*, CL9171.Contig5_All) were negatively regulated in response to 6-BA treatment. Additionally, the expression of several unigenes that involved in the regulation of hormonal signaling of ethylene (CL1635.Contig5_All, CL8680.Contig2_All, CL15911.Contig2_All and CL2328.Contig15_All), gibberellin (CL3574.Contig42_All, CL13937.Contig2_All and CL13937.Contig3_All) and jasmonic acid (CL2992.Contig3_All, CL1493.Contig4_All and CL1493.Contig2_All) was also altered in response to 6-BA treatment (Fig. 7). These results suggested that cytokinin might regulate the floral development via interactions with the other phytohormones in *S. sebiferum*.

### Identification of the differentially expressed genes related to the floral development

**Fig. 8:**
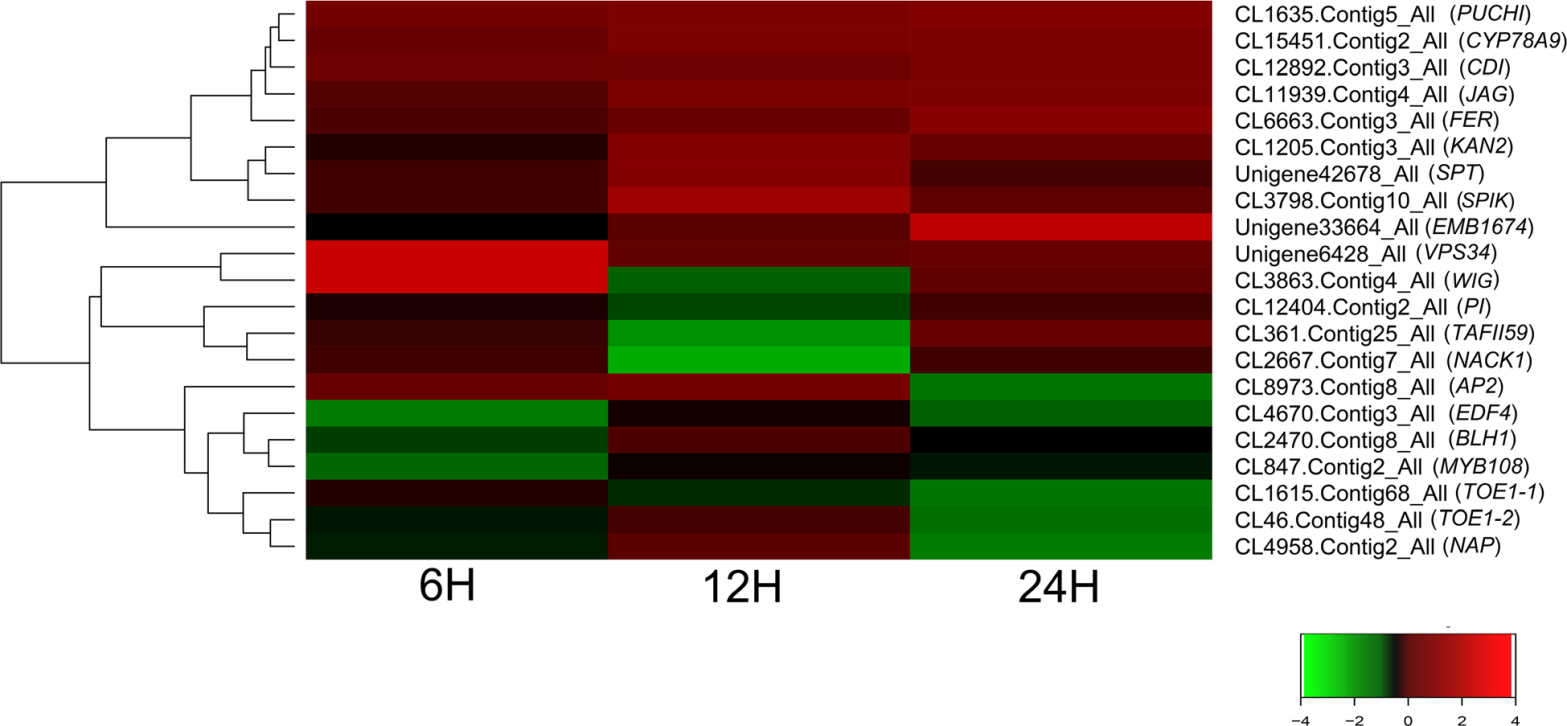
Hierarchical clustering of the differentially expressed genes involved in the regulation of floral development and floral organ identity at 6 h, 12 h and 24 h after 6-BA treatment in *S. sebiferum*

The floral organ identity genes that were differentially regulated after 6-BA treatment could directly contribute to the altered sex differentiation in *S. sebiferum*. The transcriptome data showed that a total of 21 unigenes that closely related to flowering were found to be differentially expressed in response to 6-BA treatment (Fig. 8). Five genes that related to the early floral meristem development, such as *PUCHI* (CL1635.Contig5_All), *Target of Early Activation Tagged 1* (*TOE1*, CL1615.Contig68_All and CL46.Contig48_All), *Ethylene Response DNA Binding Factor 4* (*EDF4*, CL4670.Contig3_All) and *WIGGUM* (*WIG*, CL3863.Contig4_All), were differentially regulated by 6-BA treatment (Fig. 8). Further, several key genes that directly regulated the gynoecium development, such as *SPATULA* (*SPT*, Unigene42678_All), *KANADI2* (*KAN2*, CL1205.Contig3_All), *JAGGED* (*JAG*, CL11939.Contig4_All), *Cytochrome P450 78A9* (*CYP78A9*, CL15451.Contig2_All), and *APETALA2* (*AP2*, CL8973.Contig2_All) were differentially regulated at 6 h, 12 h or 24 h after 6-BA treatment (Fig. 8). Moreover, a series of unigenes such as *MYB Domain Protein 108* (*MYB108*, CL847.Contig2_All), *Cadmium Induced* (*CDI*, CL12892.Contig3_All), *NPK1-Activating Kinesin 1* (*NACK1*, CL2667.Contig7_All), *Shaker Pollen Inward K+ Channel* (*SPIK*, CL3798.Contig10_All), *Vacuolar Protein Sorting 34* (*VPS34*, Unigene6428_All), *TATA Box Associated Factor II 59* (*TAFII59*, CL361.Contig25_All), *FERONIA* (*FER*, CL6663.Contig3-All), *Embryo Defective 1674* (*EMB1674*, Unigene33664_All), *PISTILLATA* (*PI*, CL12404.Contig2_All), which were related to the development of the male organs, were also differentially expressed after 6-BA treatment (Fig. 8). Conclusively, the identification and characterization of the cytokinin-responsive DEGs related to the floral organ identities provided insights in understanding the sex determination in *S. sebiferum*.

## Discussion

### Cytokinin has strong feminization effects on the flower development in both monoecious and androecious genotypes of *S. sebiferum*

The regulation of the female to male flower ratio is critical for improvement of the seed yield in the monoecious plants. Various phytohormones were demonstrated to be important regulators not only in vegetative growth, but also in the flower initiation and sex differentiation. In plants, phytohormones such as ethylene [29], gibberellins [30] and auxins [5, 31], have feminization effects in various species. Cytokinins are essential for numerous decisions throughout the plant developmental processes and adaptation to the biotic and abiotic environment [32]. In *Jatropha curcas*, *Plukenetia volubilis* and *Luffa cylindrica*, exogenous CK treatment can increase the number of female flowers [9, 10, 33]. Our present study in *S. sebiferum* were consistent with these findings that 6-BA or TDZ treatment has strong feminization effects on the floral development (Fig. 1). Although tZ and kinetin were two other types of cytokinins that can induce the division of plant cells [34], exogenous application with tZ and kinetin had no effects on the floral development in *S. sebiferum* (Fig. 1f). We postulated that in *S. sebiferum*, the regulation of floral sex might be controlled by specific cytokinin types.

Our results further demonstrated that 6-BA or TDZ application can also effectively induce the female flowers on the androecious *S. sebiferum* (Fig. 3). These findings suggested that cytokinin was not only the key regulator in controlling the female flower development, but also possibly was the potential determinant that involved in the formation of the androecious plants in *S. sebiferum*. The transcriptome sequencing of the male floral buds subjected to 6-BA treatment was carried out and abundant cytokinin-responsive genes that were possibly involved in female flower induction were further identified. The floral buds of the androecious *S. sebiferum* seemed to be less sensitive to 6-BA treatment in the transcriptomic level, since the number of the differentially expressed genes in response to 6-BA treatment (especially at 6 h) was fewer (Fig. 4), compared with the transcriptome results from the other plant species [7, 35–37]. We postulated that the formation of the androecious plants in *S. sebiferum* was possibly due to the genetic mutations that caused the less-sensitivity to cytokinins. Nevertheless, exogenous 6-BA treatment can still effectively induce the female flower development on the androcious *S. sebiferum* (Fig. 3). Transcriptome analysis of the transition of the inflorescences from androecious to monoecious on the androecious plants will provide valuable information of the regulative gene network of the cytokinin-regulated sex differentiation in *S. sebiferum*.

### Cytokinin regulated the cell cycle and nutrient translocation

The originally identified function of cytokinin was to promote the cell division [38]. In this study, ten cell cycle-related genes were identified in response to 6-BA treatment in the transcriptome database (Fig. 6a). Three D-type *CYCD6* (CL3342.Contig16_All and CL3342.Contig11_All), *CYCD3* (CL6841.Contig3_All) and one H-type *CYCH1* (CL384.Contig6_All) cyclin genes that played important roles in the regulation of cell division [39], were up-regulated by 6-BA treatment (Fig. 6a). *E2F Transcription Factor 3* (*E2F3*) was a key component in the cyclin D/retinoblastoma/E2F pathway, was also up-regulated at 24 h after 6-BA treatment [40]. *Origin of Replication Complex 1B* (*ORC1B*) that regulates the initiation of DNA replication [41], was up-regulated at 24 h after 6-BA treatment (Fig. 6a). *NEDD1* encoding a WD40 repeat protein that plays a critical role in the cell mitosis in interactions with tubulin complex [42], was significantly up-regulated by 6-BA treatment (Fig. 6a). Two other transcription factors *Block of Cell Proliferation 1* (*BOP1*) and *NAC Domain Containing Protein 68* (*NAC068*) that involved in the cell cycle regulation [43, 44], were also up-regulated in response to 6-BA (Fig. 6a). Additionally, several genes encoding the DNA/RNA polymerases, such as *Nuclear RNA Polymerase C1* (*NRPC1*, CL2915.Contig1_All), *Poly*(*ADP-Ribose*) *Polymerase 1* (*PARP1*, CL8049.Contig5_All), *NRPB4* (CL4312.Contig3_All) and *Poly*(*A*) *Polymerase 2* (*PAPS2*, CL14256.Contig6_All), were also up-regulated by 6-BA treatment (Fig. 6a). These results suggested that exogenous application with cytokinin could cause immediate cell replication and organ enlargement via regulating the cell-cycle related genes in the floral buds in *S. sebiferum*.

Further, a few genes that involved in the translocation of the nutrients, such as sugar, peptide, magnesium, ammonium, nucleotide, calcium, zinc and phosphate, were also differentially expressed in response to 6-BA treatment (Fig. 6b). The altered nutrients transportation to the floral bud after 6-BA treatment could be associated with the vigorous female flower induction in the floral bud. It has been demonstrated that sugars, such as sucrose, glucose and trehalose-6-phosphate were the initiation signals involved in the flower initiation in *Arabidopsis* [23]. Trehalose-6-Phosphate Synthases (TPS) were the key enzymes involved in trehalose-6-phosphate biosynthesis. The homologs of *TPS1* in *S. sebiferum* were up-regulated, whereas *TPS9* and *TPS10* were down-regulated in response to 6-BA treatment (Fig. 6b). Several genes such as *Early Response to Dehydration 6* (*ERD6*), *Glucose-6-Phosphate Translocator 2* (*GPT2*), *Nucleotide/Sugar Transporter* (*NST*) that involved in the sugar translocation [45–47], were also differentially regulated by 6-BA treatment (Fig. 6b). These results suggested that the cytokinin-responsive cell cycle and cell metabolism-related genes could be involved in the regulation of flowering.

### Cytokinin regulated the biosynthesis, transportation and signaling of other phytohormones in the regulation of sex determination in *S. sebiferum*

Phytohormones, such as auxin, cytokinin (CK), abscisic acid (ABA), ethylene (ETH), brassinosteroid (BR), gibberellin (GA), jasmonic acid (JA) and salicylic acid (SA), coordinate plant growth and development by modulating various cellular changes in response to the environmental or intrinsic signals [4]. The transcriptome data showed that many genes that were directly related to the biosynthesis, transportation and signaling of other phytohormones, were differentially regulated by 6-BA treatment in the floral buds of the androecious *S. sebiferum*. Specifically, ten genes were identified in auxin biosynthesis and signaling, eight genes in ABA biosynthesis and signaling, four genes in ethylene signaling, three gene in GA signaling, and three genes in JA signaling (Fig. 7). Previous studies demonstrated that cytokinin and auxin mutually regulated their biosynthesis and signaling in the regulation of plant growth and development [48]. Exogenous application with cytokinin in young shoot up-regulated the expression of auxin biosynthesis genes, such as *YUCCA* (*YUC*) [49]. In this study, two *YUC* genes *YUC2* and *YUC4* were found to be significantly up-regulated at 6 h, 12 h and 24 h in response to 6-BA treatment (Fig. 7). *Ring Domain Ligase 2* (*RGLG2*) was previously reported to be involved in the regulation of auxin metabolism. Mutation of *RGLG2* could cause significant decrease of auxin content in both leaf and root in *Arabidopsis* [50]. The expression of *RGLG2* was increased in response to 6-BA treatment in *S. sebiferum* (Fig. 7). The results also showed that the expression of *Dioxygenase for Auxin Oxidation 1* (*DAO1*), which encoded a major IAA oxidase in plants [28], was found to be down-regulated at 12 h, whereas promoted at 24 h (Fig. 7). These results suggested that the auxin biosynthesis was transiently promoted, at least in a short time after 6-BA treatment at the floral buds. Additionally, several auxin signaling and transportation-related genes, such as *Auxin Response Factor 2* (*ARF2*), *Indole-3-Acetic Acid Inducible 13* (*IAA13*), *Indole-3-Acetic Acid Inducible 26* (*IAA26*), *GH3.9*, *Auxin Resistant 3* (*AXR3*) and *Protein Phosphatase 2A* (*PP2A*) were found to be mostly up-regulated in response to 6-BA treatment (Fig. 7). It has been demonstrated that auxin was important for the female reproductive organ development in *Arabidopsis* [51], and also exhibited a strong feminization effect on *Cannabis sativus* and *Opuntia stenopetala* [5, 31]. The expression of auxin signaling genes was also positively correlated with the transition to female flowers on the monoecious *Ricinus communis* [52]. Our results were in accord with these findings that auxin could also play important role in the female flower induction regulated by cytokinin in *S. sebiferum*.

In cucumber, ABA did not change the sex expression, nevertheless it promoted the female tendency of gynoecious plants [53]. *XERICO* and *Nine-cis-epoxycarotenoid Dioxygenase 3* (*NCED3*) are two important regulators in ABA biosynthesis [54, 55]. The transcriptome data showed that these two genes were significantly down-regulated by 6-BA treatment (Fig. 7). The ABA signaling-related genes, such as *Ring Domain Ligase 2* (*RGLG2*), *ATMYB44* (*MYB44*), *Growth Regulating Factor 3* (*GRF3*) and *Abscisic Acid Responsive Elements-binding Factor 3* (*ABF3*) were down-regulated by 6-BA treatment (Fig. 7). Previous studies showed that ABA and cytokinin antagonistically regulated many aspects of plant growth and development [56, 57]. In *S. sebiferum*, the cytokinin responsiveness of the ABA biosynthesis and signaling-related genes suggested an antagonistic interactions between CK and ABA in the regulation of the floral development.

**Fig. 9:**
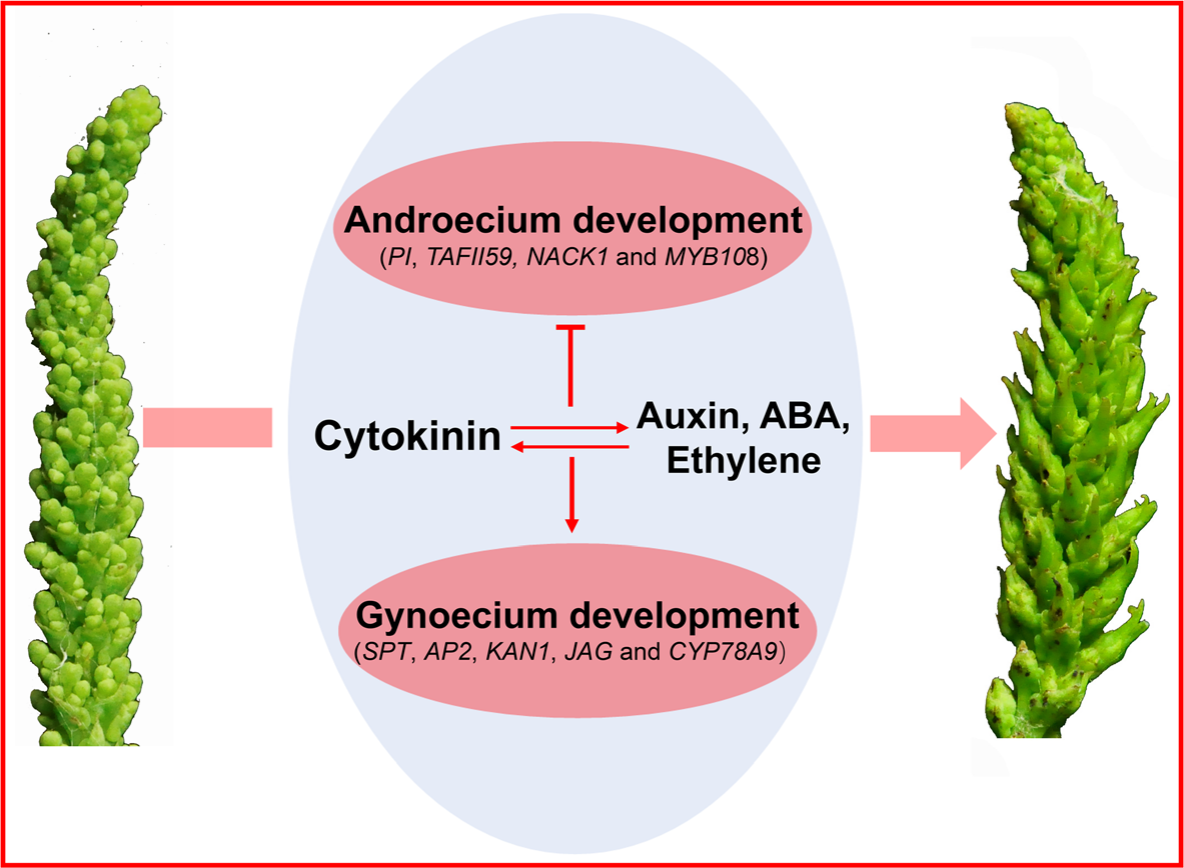
Role of cytokinins in the regulation of sex determination in *S. sebiferum*

Cytokinin also interacted with ETH, GA and JA in the regulation of flowering [58]. *PUCHI*, a key component in ethylene-activated signaling pathway, was required for morphogenesis in the early floral meristem growth [59]. The transcript abundance of *PUCHI* in the floral bud was up-regulated in response to 6-BA treatment (Fig. 7). The *S. sebiferum* orthologs of three additional ethylene signaling-related genes, *Ethylene Response Factor 74* (*ERF74*), *Ethylene and Salt Inducible 3* (*ESE3*) and *Transcription Factor IID* (*TFIID*) were also differentially regulated by 6-BA treatment (Fig. 7), suggesting a potential interactions between CK and ETH in the regulation of sex determination in *S. sebiferum*. Among these differentially expressed genes after 6-BA treatment, three JA signaling genes *ATMYC2* (*MYC2*), *Jasmonate Insensitive 1* (*JAI1*), *BHLH14* and three GA signaling genes *GA-Stimulated Arabidopsis 1* (*GASA1–1* and *GASA1–2*) and *Glabrous Inflorescence Stems 2* (*GIS2*) were differentially regulated in response to 6-BA treatment (Fig. 7). Conclusively, these results implied that cytokinin interacted with auxin, ABA, ethylene, JA and GA in sex determination in *S. sebiferum* (Fig. 9).

### Cytokinin regulated the expression of gynoecium and androecium identity-related genes

6-BA treatment profoundly induced the formation of female flowers in the androecious *S. sebiferum* (Fig. 3). The differential expression of ABCE floral organ-identity genes has been implicated in the regulation of male and female flower development [60, 61]. Thus it suggested that cytokinin could directly target the floral organ-identity genes in the regulation of sex determination. A total of 21 flowering-related genes were identified in response to 6-BA treatment at the floral bud of the androecious plants (Fig. 8). Several early floral meristem growth-related genes, such as *PUCHI*, *Target of Early Activation Tagged 1* (*TOE1*), *Ethylene Response DNA Binding Factor 4* (*EDF4*) and *WIGGUM* (*WIG*), were differentially regulated in response to 6-BA treatment (Fig. 8), suggesting cytokinin was directly involved in the regulation of the early floral meristem development in *S. sebiferum*. Among the DEGs, a few genes that were closely related to the gynoecium and androecium development, were found to be differentially regulated in response to 6-BA treatment. *SPATULA* (*SPT*), a *bHLH* transcription factor, was involved in gynoecium development [62], was up-regulated at 12 h after 6-BA treatment (Fig. 8). *KANADI 2* (*KAN2*), which encodes a nuclear-localized protein involved in the development of the carpel and the outer integument of the ovule [63], was also up-regulated at 12 h (Fig. 8). The transcript abundance of two other genes *JAGGED* (*JAG*) and *Cytochrome P450 78A9* (*CYP79A9*)*,* which were positively related to gynoecium development [64, 65], were up-regulated at 12 and 24 h (Fig. 8). *APETALA 2* (*AP2*), a member of the *AP2*/*EREBP* class of transcription factors, was involved in the specification of floral organ identity. In *Arabidopsis*, mutation of *AP2* would cause the transition of perianth organs to carpels [66]. The transcriptome results showed that the expression of *AP2* in *S. sebiferum* was significantly decreased at 24 h after 6-BA treatment (Fig. 8), suggesting a feminization effect of cytokinin on the floral sex in *S. sebiferum*. Additionally, several genes, such as *MYB Domain Protein 108* (*MYB108*), *NPK1-Activating Kinesin 1* (*NACK1*), *TATA Box Associated Factor II 59* (*TAFII59*) that were important in the regulation of androecium development [67–69], were down-regulated in response to 6-BA treatment (Fig. 8). *PISTILLATA* (*PI*) which encodes a MADS domain transcription factor in regulating the specification of the petal and stamen identities [70], was also down-regulated by 6-BA treatment (Fig. 8), suggesting cytokinin had inhibitory effects on the androecium development in *S. sebiferum*. These results demonstrated that cytokinin regulated the sex differentiation in *S. sebiferum* by directly targeting the genes related to the gynoecium and androecium development (Fig. 9).

## Conclusions

Conclusively, this study revealed that exogenous application with cytokinin has strong feminization effects on the floral development in *S. sebiferum*. The transcriptome analysis of the transition from the androecious to monoecious inflorescences after 6-BA treatment on the androecious *S. sebiferum* contributed to the identification of the candidate genes that were involved in the regulation of sex determination in response to cytokinin. Our study provided a basis for further understanding the molecular mechanisms of the hormonal regulation of sex determination in *S. sebiferum*.

## Additional files

Additional file 1: **Figure S1.** Photographs of the floral buds used for hormonal treatment and RNA-sequencing. (TIF 5697 kb)

Additional file 2: **Table S1.** Primers for quantitative real-time PCR (DOCX 16 kb)

Additional file 3: **Figure S2.** Photograph of the inflorescences of different genotypes of *S. sebiferum*. (TIF 1797 kb)

Additional file 4: **Tables S2-S4.** Information of the assembled genes at 6 h, 12 h and 24 h. (XLSX 48555 kb)

Additional file 5: **Tables S5-S7.** Differentially expressed genes at 6 h, 12 h and 24 h after 6-BA treatment. (XLSX 363 kb)

Additional file 6: **Figure S3.** GO analysis of the differentially expressed genes at 6 h (A), 12 h (B) and 24 h (C) after 6-BA and mock treatment. (TIF 1842 kb)

## Abbreviations

ABA: Abscisic acid
BA: Benzylaminopurine
DEG: Differentially expressed genes
ETH: Ethylene
GA: Gibberellin
GO: Gene ontology
JA: Jasmonic acid
KEGG: Kyoto Encyclopedia of Genes and Genomes
PCR: Polymerase chain reaction
qPCR: Quantitative real time polymerase chain reaction
RNA-seq: RNA sequencing
RPKM: Reads per kilobase per million mapped reads
TDZ: Thidiazuron
TF: Transcription factor
