# Supplementary figures and images for: Comparative transcriptome analysis reveals the regulatory networks of cytokinin in promoting the floral feminization in the oil plant Sapium sebiferum
Source: BMC Plant Biol. 2018 May 30;18:96. doi: 10.1186/s12870-018-1314-5 (PMC5975670; doi:10.1186/s12870-018-1314-5)

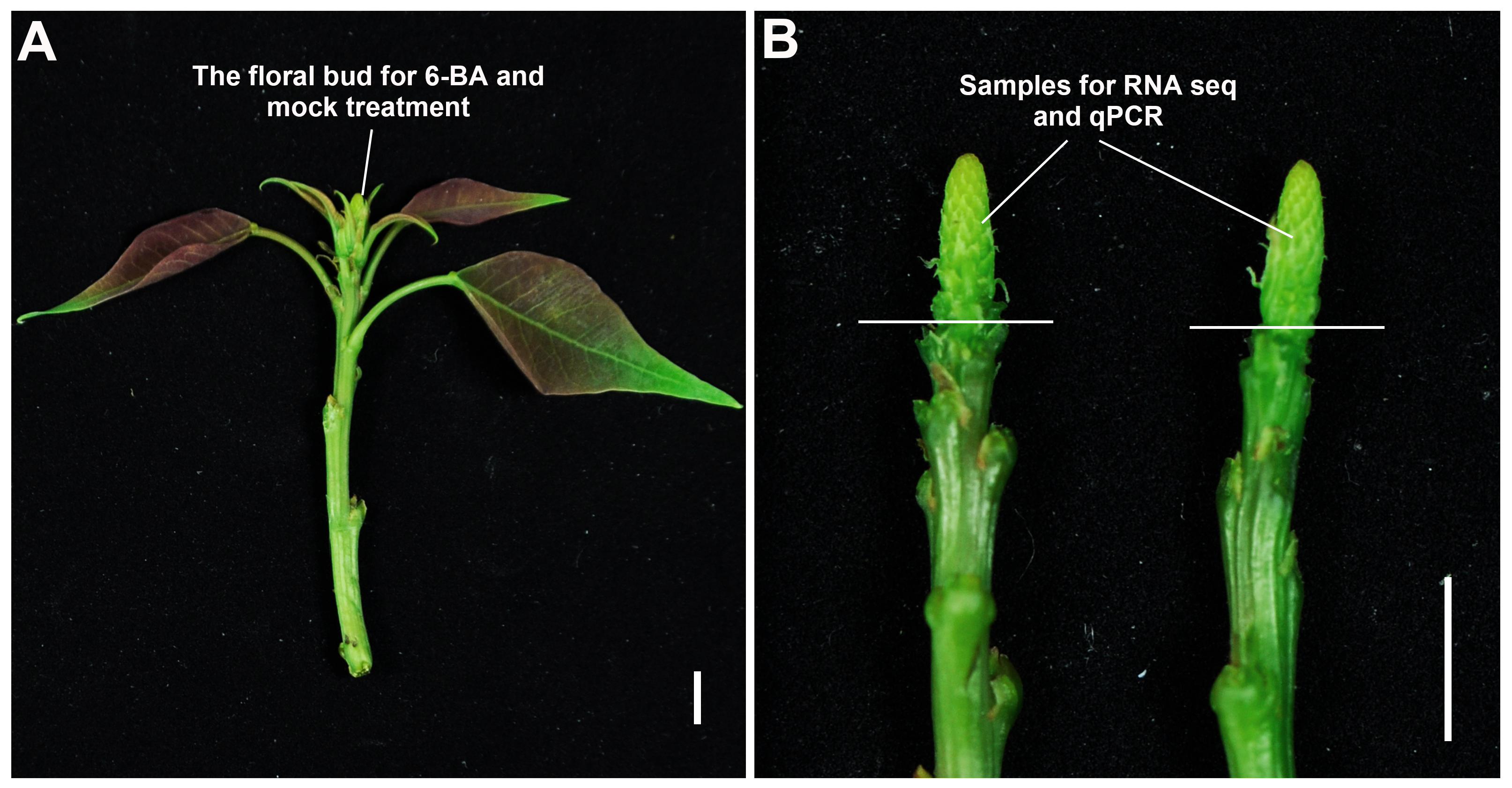

Supplement: Supplementary file 1 — Figure S1. Photographs of the floral buds used for hormonal treatment and RNA-sequencing. (TIF 5697 kb) [file 12870_2018_1314_MOESM1_ESM.tif]

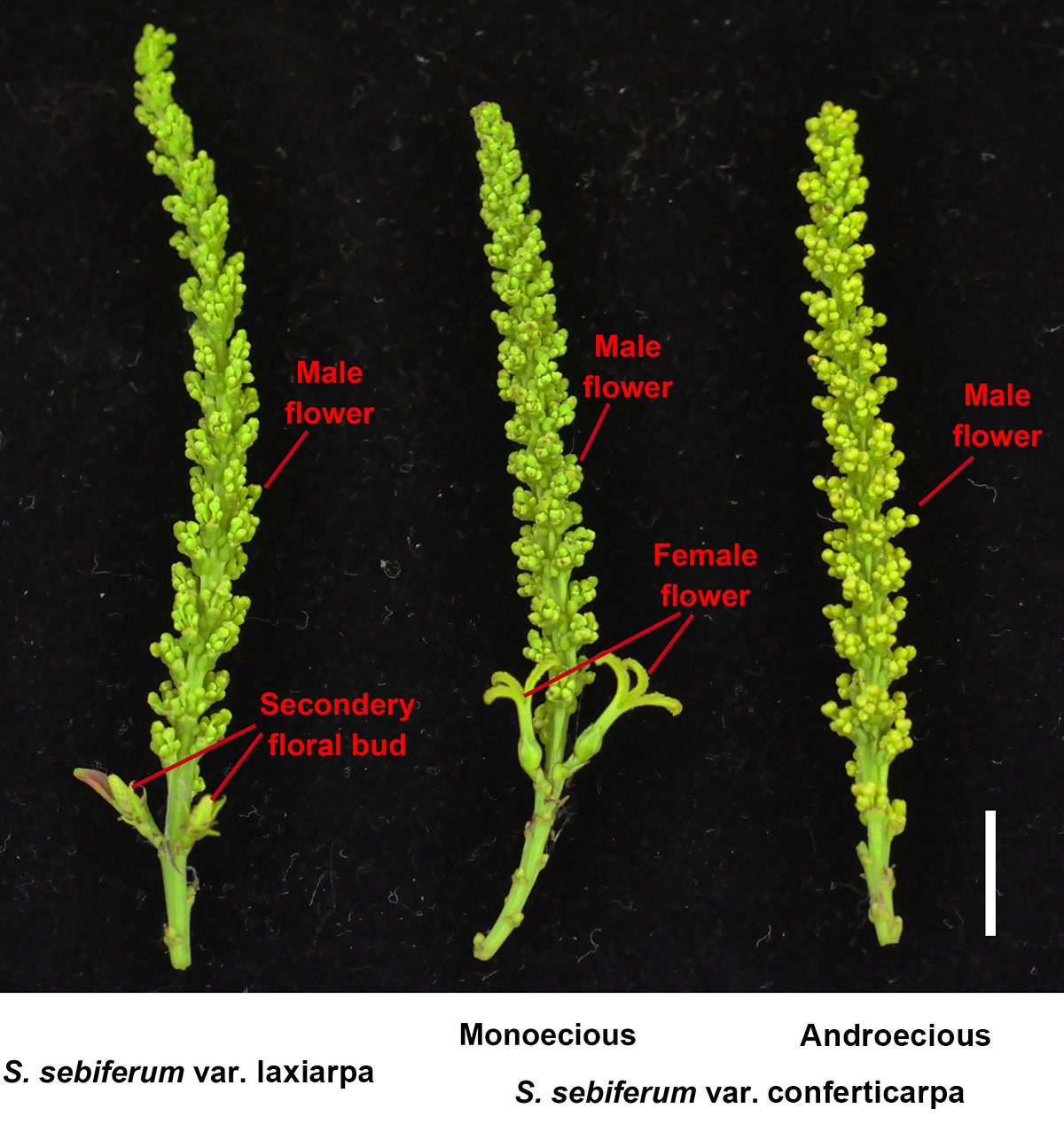

Supplement: Supplementary file 3 — Figure S2. Photograph of the inflorescences of different genotypes of S. sebiferum. (TIF 1797 kb) [file 12870_2018_1314_MOESM3_ESM.tif]

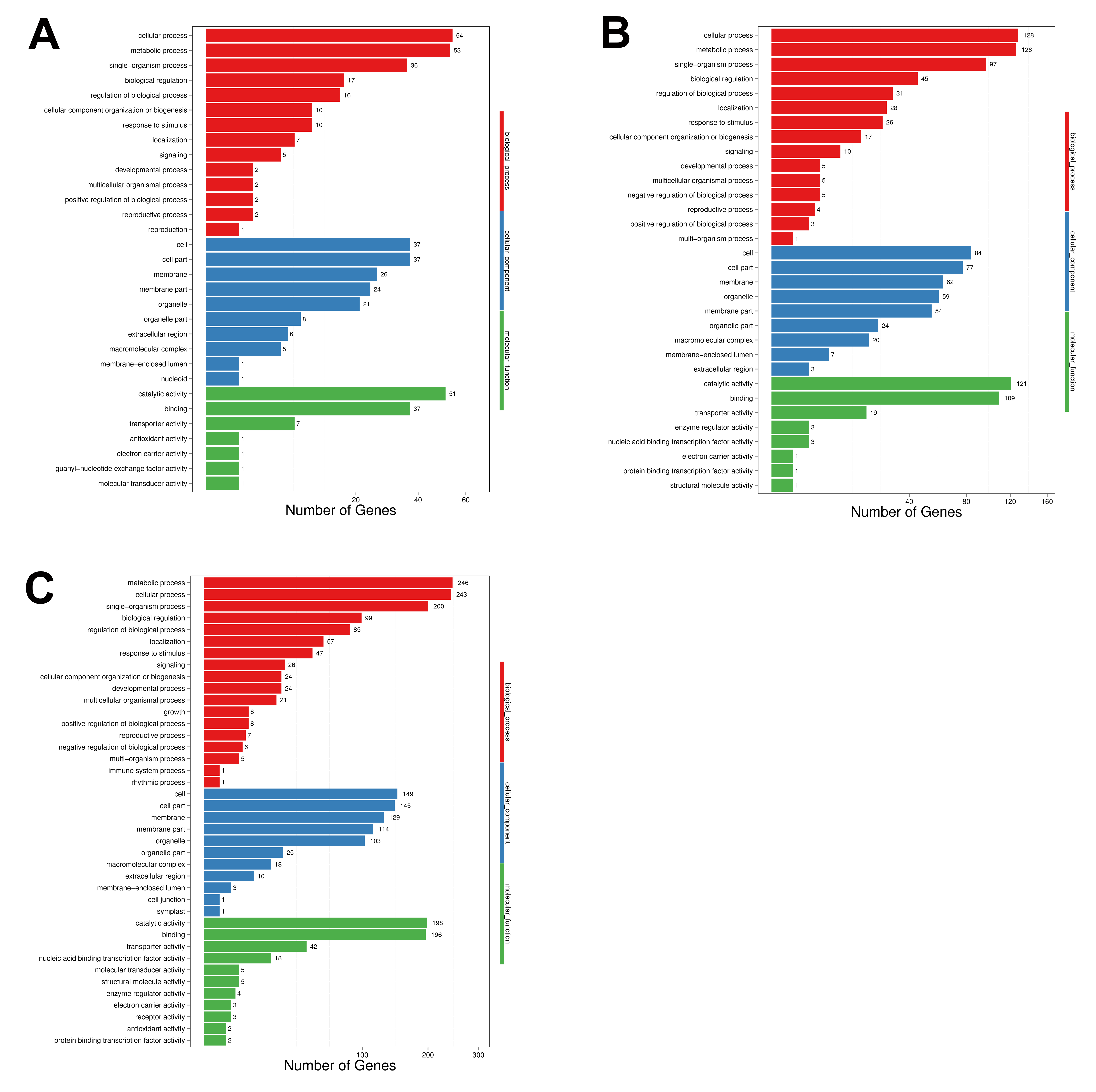

Supplement: Supplementary file 6 — Figure S3. GO analysis of the differentially expressed genes at 6 h (A), 12 h (B) and 24 h (C) after 6-BA and mock treatment. (TIF 1842 kb) [file 12870_2018_1314_MOESM6_ESM.tif]
