# Supplementary material for: Comparative transcriptome analysis reveals the regulatory networks of cytokinin in promoting the floral feminization in the oil plant Sapium sebiferum
Source: BMC Plant Biol. 2018 May 30;18:96. doi: 10.1186/s12870-018-1314-5 (PMC5975670; doi:10.1186/s12870-018-1314-5)
Supplement: Supplementary file 2 — Table S1. Primers for quantitative real-time PCR (DOCX 16 kb) [file 12870_2018_1314_MOESM2_ESM.docx]

Table S1. Primers for quantitative real-time PCR.

| Gene ID | Direction | Primer sequence |
| --- | --- | --- |
| CL3798.Contig10_All | Sense | TAGAAGGAGGACTACACAATGCTCACT |
|  | Antisense | TGATCTGTTGCTGCTTCAGTTGCTTA |
| CL15451.Contig2_All | Sense | CTAAGGAGAATCGCAGCAACTCATCTT |
|  | Antisense | ACCGAGCACATCATGTTGTTAAGTGA |
| CL14312.Contig3_All | Sense | GCAGCTTCAACAGATGTCTGAGGAT |
|  | Antisense | CTCCACAGTTTCAGGGCAAAGGTT |
| CL10984.Contig2_All | Sense | TCCTATGCTGCTCACTTCTCTATCCAA |
|  | Antisense | TTCACCAGTAGCAACAATAAGCCAAGA |
| CL8402.Contig2_All | Sense | CTTCACCAAAGCCTCCATTCTCTCAA |
|  | Antisense | TCAAGGAAGCTCCATCCACCGATAT |
| CL1856.Contig26_All | Sense | GCTCCACACTGCTGTCAGGTTG |
|  | Antisense | TGTCTTGTTCTTGCTGCTACAGGATC |
| CL1323.Contig24_All | Sense | GGACTGTTACATTAGCACTGGTGATGA |
|  | Antisense | GAGATTCTCGGCATGGACTTCTGATAC |
| CL14183.Contig4_All | Sense | GAATAGCAAGCAATGGATGAACGACAA |
|  | Antisense | ATCAACTGGAAGACATTGAGCATAGCA |
| CL4361.Contig13_All | Sense | AACAGAACATAGCCAAGTGACTCCAAA |
|  | Antisense | GAGATGCCACAACCAATCGCCATA |
| CL4670.Contig3_All | Sense | CTCCATCTTGCGGCTTGAAGTTAGT |
|  | Antisense | TACGAGAAGCACCAGCGAGTGT |
| CL8816.Contig1_All | Sense | GCTCCAAGGCATTGTCTCCTGAAT |
|  | Antisense | ACCTCTTCCAGTTCCTCCAGTGTT |
| CL2483.Contig2_All | Sense | GCTTACATCCGCTGCATGAAGTCT |
|  | Antisense | GTGCCTGAGATGACACAGATACGATC |
| CL7852.Contig4_All | Sense | AACACGGTGCCGAACATGACATT |
|  | Antisense | ATCTTCATTATCACCTTCGCCTTCTCC |
| CL14906.Contig2_All | Sense | CAATCTCTGTCTTCTCAACTG |
|  | Antisense | CTCTATGACCTATGTGTTCCA |
